# Association between physical activity and major adverse cardiovascular events in northwest China: A cross-sectional analysis from the Regional Ethnic Cohort Study

**DOI:** 10.3389/fpubh.2022.1025670

**Published:** 2022-11-17

**Authors:** Yutong Wang, Huimeng Liu, Dandan He, Binyan Zhang, Yezhou Liu, Kun Xu, Suixia Cao, Yating Huo, Jingchun Liu, Lingxia Zeng, Hong Yan, Shaonong Dang, Baibing Mi

**Affiliations:** Department of Epidemiology and Biostatistics, School of Public Health, Xi'an Jiaotong University Health Science Center, Xi'an, Shaanxi, China

**Keywords:** physical activity, cardiovascular disease, metabolic equivalent task, dose-response relationship, regional health

## Abstract

**Background:**

To examine the association between daily physical activity (PA) and major adverse cardiovascular events (MACEs) in northwest China.

**Methods:**

The data in this analysis were part of the baseline survey of the Regional Ethnic Cohort Study in Northwest China from June 2018 to May 2019 in Shaanxi Province. This study used standardized self-reported total physical activity (continuous and categorical variables) and self-reported outcomes of MACEs. All analyses were conducted using the logistic regression model and stratified by age, sex, body mass index (BMI), and region. The dose-response relationships were assessed with a restricted cubic spline.

**Results:**

The average level of total PA was 17.60 MET hours per day (MET-h/d). Every increase of four MET-h/d of total PA was associated with a lower risk of MACEs [adjusted OR = 0.95 (95% CI, 0.93~0.98)]. Compared with participants in the bottom quartile of total PA, a lower risk of MACEs was observed in the top quartile group [≥23.3 MET-h/d, 0.68 (0.55~0.83)]. Stratified analyses showed similar results in males, females, participants over 45 years old, participants in the rural region, and normal weight range participants (BMI < 24 kg/m^2^). Total participants also observed a dose-response relationship after adjusting for socioeconomic and lifestyle factors.

**Conclusions:**

A higher level of PA was associated with a lower MACE risk. Future research should examine the longitudinal association of prospectively measured PA and the risk of MACEs.

## Introduction

Major adverse cardiovascular events (MACEs) have been used in cardiovascular disease (CVD) research, with MACEs selected as the primary or secondary endpoint (1). Physical activity (PA) is an easily modifiable lifestyle factor that is widely recommended due to its demonstrated beneficial effect on health outcomes (2, 3), both physically (4) and psychically (5–7). It is also an essential behavioral risk factor for CVD (8). A cohort study in China demonstrated that the population attributable risk of major coronary events due to lack of PA was 21.6% (9). In addition, a meta-analysis showed that a high level of leisure-time PA and a moderate level of occupational PA have beneficial effects on cardiovascular health (10).

The prevalence of a high risk of CVD illustrated a regional disparity in China, especially in the northern region (9.6% in the northwest region, 12.6% in the northeast region, and 8.0% in the south region) (11). Additionally, the disease patterns in China differ notably from those in high-income countries (e.g., there were higher rates of stroke than ischemic heart disease in China) (12, 13) and vary significantly between regions (e.g., stroke was more common in the eastern region, and myocardial infarction was more common in the central and western regions) (8). Most previous studies on the association between PA and the risk of MACEs were based on studies conducted in high-income countries (14–17), lacking evidence from China. A prospective cohort study in 10 areas across China found that every increase of four metabolic equivalents of task (MET) hours per day reduced the risk of major vascular events by 6% (18). A prospective study in the United States showed that each MET improvement in midlife was associated with a 17% lower risk of heart failure hospitalization in later life (19).

In addition, the patterns of PA, including the domains and intensity of physical activities, appear to vary greatly across different regions in China (20). A prior study showed that the prevalence of leisure-time PA in adults was also lower in undeveloped western areas than in central and eastern China (21). Therefore, studies focused on specific regions with distinctive characteristics, such as relatively poor financial terms, varied regional topography, and ethnic diversity (22), are warranted.

Based on data from the baseline surveys of a large prospective study in northwest China, this study aimed to quantify the relationship between PA and MACE risk in Shaanxi Province and assess whether the association differed by sex, age, body mass index (BMI), and region. Besides, considering that people with diabetes have specific features that lead them to higher CVD risk compared with the general population, we further test the association between total PA and the risk of MACE in the participants with and without diabetes as an exploratory analysis.

## Materials and methods

### Study design and study population

The analyses were based on data from the Regional Ethnic Cohort Study in Northwest China (RECS), a community population-based prospective observational study in which participants completed a baseline survey from June 2018 to May 2019. The study design, methods, and recruitment strategy have been described previously (22). All study procedures were conducted following the ethical standards of the responsible committee on social and behavioral science research and with the Helsinki Declaration of 1975, revised in 2000. This cohort study was approved by the Human Research Ethics Committee of Xi'an Jiaotong University (No: XJTU2016-411). All participants were informed and signed the informed written consent.

**Figure 1:**
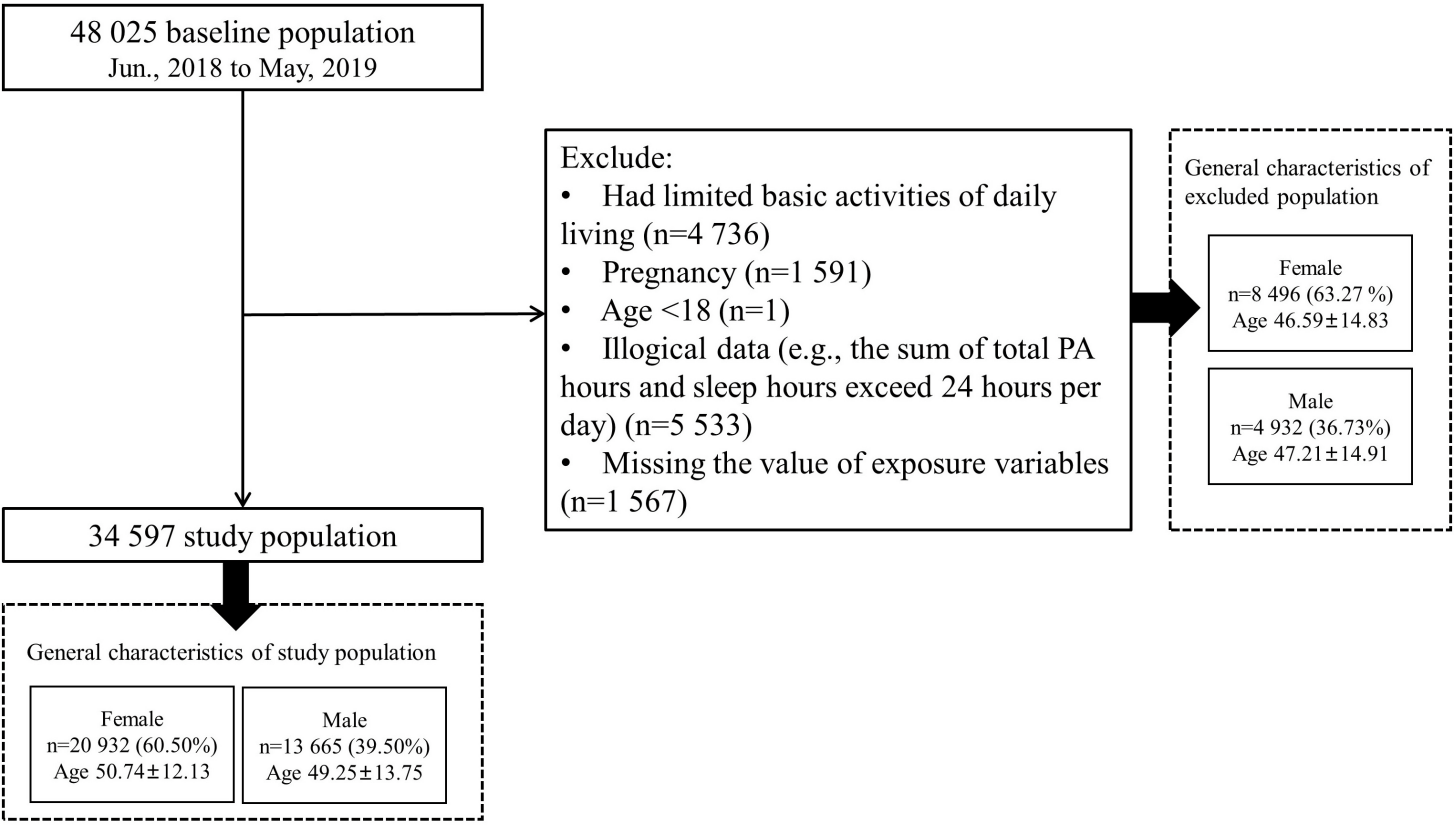
Flow diagram of the study population.

In this analysis, we focused on 48,025 participants from Shaanxi Province to explore the association between PA levels and the risk of MACEs. After data cleaning and logical verification, we excluded those participants who were pregnant (*n* = 1,591), had limited basic activities of daily living (*n* = 4,736), had illogical data (e.g., the sum of total PA hours and sleep hours exceeded 24 h per day; *n* = 5,533), were younger than 18 years old at the baseline survey (*n* = 1), and had missing exposure variables (*n* = 1,567), leaving 34,597 participants for this analysis (Figure 1).

### Physical activity measurements

The questions on physical activity in this study were in line with the questionnaire of the China Kadoorie Biobank (CKB) (18). The questionnaires were adapted from validated questionnaires used in several other studies, including high-income countries (23) and the Chinese population (24), with some additional modifications after a CKB pilot study (25). However, the questionnaires have not been compared directly with a reference method, such as an accelerometer (18).

Metabolic equivalent tasks (METs) from the 2011 update (26) of a major compendium of physical activities were used to quantify the amount of PA (Supplementary Table 1). PA has been defined as “any bodily movement produced by skeletal muscles that result in energy expenditure” >1.5 metabolic equivalents (27), including time spent in light, moderate and vigorous intensity (26). The designed questionnaire used in the baseline survey of this cohort study covered relevant questions on the intensity, frequency, and time spent on occupational tasks, commuting, household tasks, and sports activities. The MET of each activity was subsequently multiplied by the frequency and duration of physical activity and summed together to calculate total physical activity in MET hours per day (MET-h/d).

### Covariates

The participants completed a questionnaire and underwent a physical health examination, including measurements of height (cm) and weight [kg; from which body mass index (BMI) was calculated as kg/m^2^]. Based on the recommended cutoff points for Chinese adults (28), we categorized BMI into three groups: normal weight (BMI < 24 kg/m^2^), overweight (24 kg/m^2^ ≤ BMI < 28 kg/m^2^), and obesity (BMI ≥ 28 kg/m^2^).

Based on the prior studies (18, 29) and knowledge, we considered sociodemographic factors and lifestyle factors as potential confounders. To facilitate choosing confounders for adjustment, we constructed a conceptual framework to visualize relationships among the exposure, outcome, and confounders by using directed acyclic graphs (DAGs; Supplementary Figure 1) with the DAGitty program (http://dagitty.net/, version 3.0) (30). The final set of confounders that we chose to adjust contained age (continuous, years), sex (male and female), study location (categorized as six different regions), household annual income [ < 10,000, 10,000–50,000, 50,000–100,000, and ≥100,000 RMB (yuan)], and education (no formal school, primary school, middle school, and college and above). Lifestyle factors included smoking (current smoker and nonsmoker), alcohol consumption (current drinker and nondrinker), sedentary leisure time (continuous, hours per day), fruit intake (every day, 4–6 times per week, 1–3 times per week, 1–3 times per month, and never intake fresh fruit), and self-reported general health status (excellent, good, fair, and poor).

### Outcomes

The primary outcome of this analysis was the prevalence of MACE. Given that this analysis was based on cross-sectional data, MACE was defined as stroke and nonfatal acute myocardial infarction (AMI) (1, 31). The outcomes were obtained from the following self-reported questions:

“*Are you suffering from chronic diseases? (yes/no).”*

(1) “*Have you ever had an acute myocardial infarction? (yes/no)*.”
(2) “*Do you have a stroke/minor stroke? (yes/no)*.”

We defined participants with AMI and/or stroke/minor stroke as the participants with MACE. Participants who missed both data were treated as missing data. The rest of the population was treated as non-MACE participants. AMI and stroke/minor stroke were set as secondary outcomes to explore the association between PA and CVD.

### Statistical analysis

The value of MET was categorized into four groups according to quartile: <9.0 MET-h/d, 9.0–15.2 MET-h/d, 15.3–23.2 MET-h/d, and ≥23.3 MET-h/d. Selected characteristics of the study participants were compared by quartile of total PA. Sociodemographic characteristics are presented using the means ± standard deviation (SD) for continuous variables and the frequencies (percentage) for categorical variables. ANOVA and chi-square tests were used to test differences based on the quartile of MET.

Given that MACE was rare in the whole population, we used a penalized maximum likelihood logistic regression model to reduce the possible biases (32). We estimated the odds ratios (ORs) for MACE with a quartile of total PA (both continuous and categorical variables) with a 95% confidence interval (CI). PA results are presented as the ORs per 4 MET-h/d higher total PA with the risk of MACEs. Model 1 was fitted by a crude penalized maximum likelihood logistic regression model, with only the MET value as the exposure. Model 2 was adjusted for sociodemographic factors (e.g., age, sex, study location, household annual income, and education). Model 3 was further adjusted for participants' lifestyle factors (e.g., smoking, alcohol consumption, sedentary leisure time, fruit intake, and self-reported general health status). We performed the same analysis on the secondary outcomes (AMI and stroke/minor stroke).

To explore the dose-response relationship between total PA and the risk of MACEs, we performed restricted cubic spline (RCS) with five knots (5th, 25th, 50th, 75th, and 95th) to explore the nonlinear relationship. The linearity of the dose-response association was tested using Wald tests (33). Age in the regression model using an RCS function was treated as a potential confounder, and the mean MET values were treated as reference spots. We further adjusted sociodemographic and lifestyle factors (Model 3) and excluded outliers (MET values that were outside three standard deviations from the mean). For subgroup analysis, we grouped the total sample by sex (males and females), age (years; 18–44, 45–59, and ≥60), BMI [kg/m^2^; normal weight (BMI < 24 kg/m^2^), overweight (24 kg/m^2^ ≤ BMI < 28 kg/m^2^), and obesity (BMI ≥ 28 kg/m^2^)], and region (urban region and rural region). In an exploratory analysis, we further grouped the total participants into two groups (with diabetes and without diabetes) to explore the effect of diabetes on the relationship between PA and MACE.

All statistical analyses were performed using SAS version 9.4 software (SAS Institute, Inc., Cary, NC). Penalized maximum likelihood estimation in logistic regression was conducted using SAS PROC LOGISTIC. Two-sided *p*-values < 0.05 were considered statistically significant.

## Results

Overall, the distributions of sociodemographic and lifestyle factors in the analytic sample (*n* = 34,597) were considerably different from those in the excluded sample (*n* = 13,428; Supplementary Table 2). Specifically, participants in the analytic sample had a younger age, higher BMI level, longer sedentary leisure time, and higher household annual income. They were more likely to be female, had a lower proportion of smokers and drinkers, and had poor health status.

Table 1 illustrates the sociodemographic factors at baseline and lifestyle factors stratified into four groups by MET quartile. The sample covered ages from 18 to 90 years old (the mean ± SD age was 50.15 ± 12.81). The mean ± SD BMI value was 23.80 ± 3.40 kg/m^2^, 60.50% (*n* = 20,932) were women, 65.87% (*n* = 22,790) were from rural regions, and 99.14% (*n* = 34,198) were of Han Chinese ethnicity. Individuals with higher total PA levels were younger, married, had a higher level of education, had higher levels of self-reported general health status, and were more likely to report hypertension than those with lower PA levels.

**Table 1:** Characteristics of Shaanxi Province people by levels of physical activity^a^.

**Characteristics**	**Total physical activity (MET-h/d)^b^**					***p*** **-value**
	**Total**	**<9.0**	**9.0–15.2**	**15.3–23.2**	**≥23.3**	
**No. of participants**	34,597	8,628	8,670	8,644	8,655	
**Mean ±SD**						
**Age, years**	50.15 ± 12.81	55.97 ± 11.20	49.76 ± 13.33	45.79 ± 12.85	49.10 ± 11.60	<0.001
**BMI, kg/m** ^**2**^	23.80 ± 3.40	23.80 ± 3.42	23.78 ± 3.39	23.81 ± 3.41	23.83 ± 3.40	0.801
**Physical activity–related factors**						
Total physical activity, MET-h/d	17.60 ± 11.50	5.51 ± 2.49	12.27 ± 1.68	18.87 ± 2.28	33.74 ± 8.85	<0.001
Sedentary leisure time, h/d	2.99 ± 1.72	3.11 ± 2.03	3.12 ± 1.76	2.94 ± 1.60	2.78 ± 1.41	<0.001
***n*** **(%)**						
**Demographic factors**						
Female	20,932 (60.50)	5,095 (59.05)	5,705 (65.80)	5,136 (59.42)	4,996 (57.72)	<0.001
Rural	22,790 (65.87)	7,405 (85.83)	5,383 (62.09)	3,987 (46.12)	6,015 (69.50)	<0.001
Han Chinese ethnicity	34,198 (99.14)	8,558 (99.41)	8,575 (99.16)	8,513 (98.85)	8,552 (99.17)	0.020
**Socioeconomic and lifestyle factors**						
Middle school	13,647 (39.60)	4,068 (47.29)	2,949 (34.15)	2,517 (29.25)	4,113 (47.74)	<0.001
Married	30,285 (88.04)	7,699 (89.63)	7,330 (85.09)	7,464 (86.89)	7,792 (90.56)	<0.001
Household annual income ≥100,000 ¥/year ^c^	4,631 (14.97)	462 (5.77)	1,254 (16.58)	2,056 (27.45)	859 (10.91)	<0.001
Current smoker	7,163 (20.91)	1,949 (22.77)	1,481 (17.28)	1,712 (20.03)	2,021 (23.56)	0.001
Current drinker	22,981 (66.72)	2,139 (24.90)	2,852 (33.06)	3,725 (43.31)	2,746 (31.84)	<0.001
**Self-reported conditions**						
Excellent status of health	11,261 (32.72)	2,211 (25.74)	2,891 (33.57)	3,223 (37.49)	2,936 (34.07)	<0.001
Hypertension	4,164 (37.50)	1,254 (35.42)	1,035 (37.28)	864 (35.35)	1,011 (43.13)	<0.001
Diabetes	997 (9.47)	304 (9.03)	257 (9.82)	209 (9.02)	227 (10.20)	0.281
MACE	1,048 (3.24)	420 (5.26)	265 (3.31)	173 (2.13)	190 (2.31)	<0.001

MET-h/d, metabolic equivalents of task per hour per day; SD, standard deviation; BMI, body mass index (calculated as weight in kilograms divided by height in meters squared); MACE, major adverse cardiovascular event.

a For some variables, the sum of categories was not equal to the total due to missing data.

b Continuous variables were presented as Mean ± SD and categorical variables were presented as *n* (%).

c RMB (yuan) was used to estimate household annual income.

**Figure 2:**
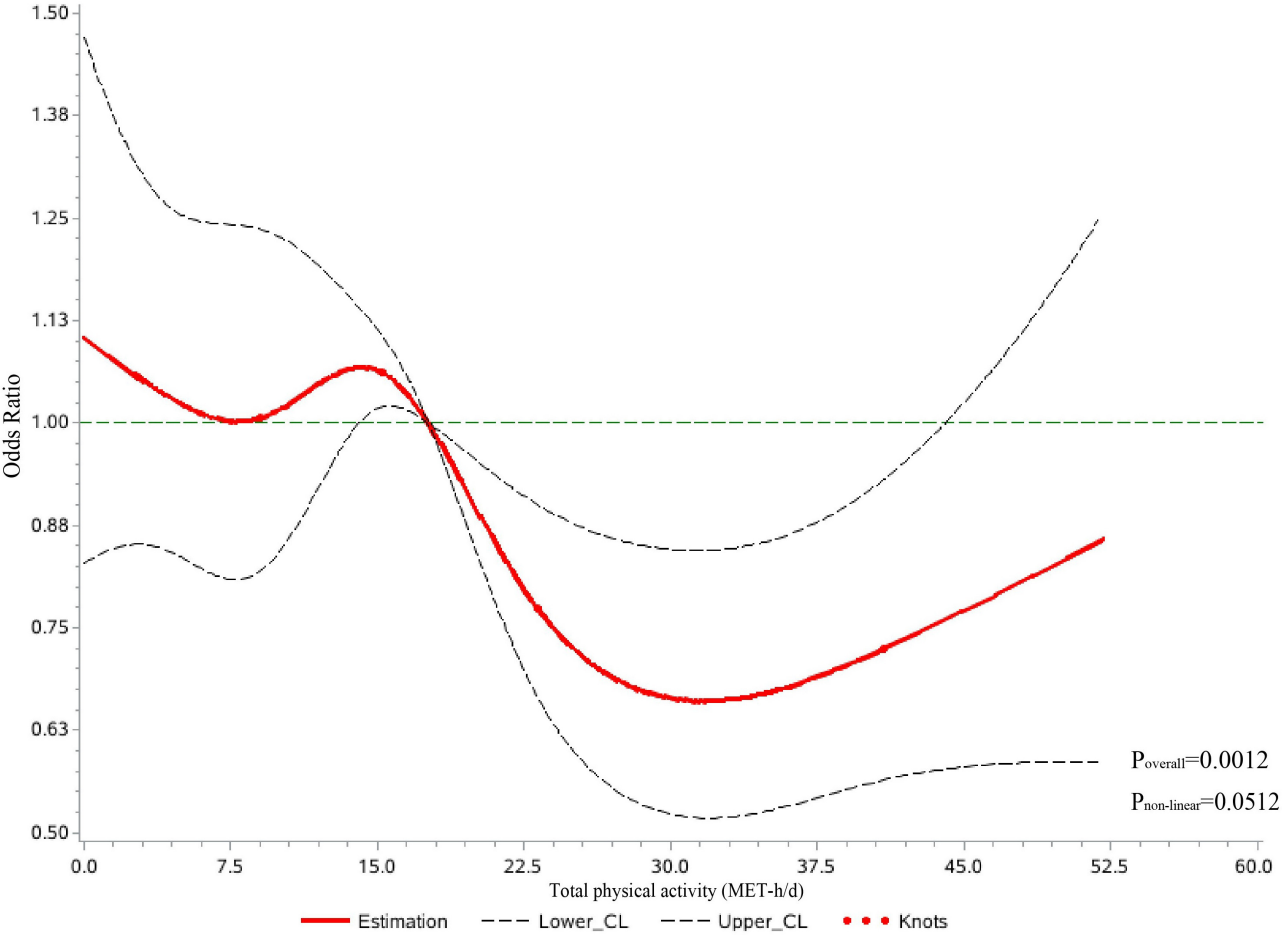
Dose-response relationship between total physical activity per day and major adverse cardiovascular events in restricted cubic spline among all participants. CL, confidence limit; MET-h/d, metabolic equivalents of task per hour per day. Curves were fitted as a smooth term using a restricted cubic spline with five knots (5th, 25th, 50th, 75th, and 95th). Age in the regression model using an RCS function was treated as a potential confounder, and the mean MET values were treated as reference spots. The model was adjusted for sociodemographic factors (e.g., sex, study location, household annual income, and education) and participants' lifestyle factors (e.g., smoking, alcohol consumption, sedentary leisure time, fruit intake, and self-reported general health status).

Table 2 shows the relationship between total PA and the risk of MACEs in all participants. Higher total PA (MET-h/d, continuous variables) was associated with a 5% lower risk of MACE [adjusted OR = 0.95 (95% CI, 0.93~0.98), *p*-value < 0.001] in every increase of four MET-h/d after adjusting for sociodemographic and lifestyle factors. For categorical variables of total PA, which were categorized into four groups by quartile, individuals in the top quartile of total physical activity were associated with a 32% lower risk of MACE [adjusted OR = 0.68 (95% CI, 0.55~0.83), *p-*value < 0.001] than those in the bottom quartile. In addition, we explored the dose-response relationship between total PA and the risk of MACEs in all participants (Figure 2). After adjustment for sociodemographic and lifestyle factors (Model 3), a higher level of total PA per day was associated with a lower risk of MACEs compared with the reference level (17.60 MET-h/d, *P*_overall_ = 0.0012, *P*_non − linear_ = 0.0512). The dose-response relationship showed a “U”-shaped association, with the 31.78 MET-h/d showing a 34% lower risk of MACEs [adjusted OR = 0.66 (95% CI, 0.52~0.84)]. Additionally, we observed the similar results in the secondary outcomes (Supplementary Table 3). Throughout the range of total physical activity studied, each four MET-h/d higher usual total PA was associated with a 9% and 4% lower risk of AMI and stroke/minor stroke, respectively. There were similar results in the total PA quartile in this study's component of MACE.

**Table 2:** Associations between total physical activity and the prevalence of MACE in all participants.

	**Sample size**	**Prevalence, *n* (%)**	**Model 1 (crude)**	***p*** **-value**	**Model 2^a^**	***p*** **-value**	**Model 3^b^**	***p*** **-value**
			**OR (95% CI)**					
PA (MET-h/d)	32,338	1,048 (3.24)	0.89 (0.87, 0.91)	<0.001	0.95 (0.93, 0.98)	<0.001	0.95 (0.93, 0.98)	<0.001
PA (Quartile)^c^								
< 9.0	7,992	420 (5.26)	1.00 [Reference]		1.00 [Reference]		1.00 [Reference]	
9.0–15.2	8,014	265 (3.31)	0.62 (0.53, 0.72)	<0.001	0.98 (0.83, 1.15)	0.782	1.03 (0.86, 1.22)	0.771
15.3–23.2	8,118	173 (2.13)	0.39 (0.33, 0.47)	<0.001	0.89 (0.73, 1.08)	0.223	0.88 (0.72, 1.08)	0.233
≥23.3	8,214	190 (2.31)	0.43 (0.36, 0.51)	<0.001	0.67 (0.56, 0.82)	<0.001	0.68 (0.55, 0.83)	<0.001

MACE, major adverse cardiovascular event; MET-h/d, metabolic equivalents of task per hour per day; OR, odds ratio; CI, confidence interval.

a Model 2 adjusted for age, sex, study location, household annual income, and education.

b Model 3 additionally adjusted for smoking, alcohol consumption, sedentary leisure time, fruit intake, and self-reported general health status.

c The value of METs was categorized into four groups by quartile: 9.0 (quartile1), 15.2 (quartile2), 23.2 (quartile3).

In the subgroup analysis, we observed similar results in males, females, participants aged over 44 years old, those who had normal weight (BMI < 24 kg/m^2^), and participants who lived in the rural region (Supplementary Tables 4–7). Specifically, in different sex groups, individuals in the top quartile of total physical activity were associated with a 27% lower risk of MACE [adjusted OR = 0.73 (95% CI, 0.56~0.97), *p*-value = 0.028] and a 38% lower risk of MACE [adjusted OR = 0.62 (95% CI, 0.46~0.82), *p*-value = 0.001] than those in the bottom quartile in males and females, respectively. However, the association between continuous variables of total PA and the risk of MACEs was only marginally significant in males [adjusted OR = 0.95 (95% CI, 0.94~1.00), *p*-value = 0.045] after adjusting for sociodemographic and lifestyle factors. In different age groups, participants over 44 years old (including 45–59 years old and over 60 years old) showed a lower risk of MACEs with the increasing daily PA, similar to the normal-weight individuals' risk. For overweight participants, we only observed a 32% lower risk of MACEs when comparing the top quartile with the bottom quartile of total PA. We further observed the relationship between total PA and lower risk of MACE in participants who lived in the rural region. In contrast, there was no significant association among urban region individuals. The association between PA and MACE was observed in participants without diabetes at the baseline survey, both in continuous and categorical variables of PA, but not in participants with diabetes (Supplementary Table 8).

## Discussion

Physical activity (PA) has been considered a simple, low-cost, and widely applicable approach to preventing MACEs (34). With data from 34,597 individuals from the baseline survey of the cohort study in Shaanxi Province, we observed that higher total PA per day was related to a lower risk of MACEs in all participants. The subgroup analyses showed similar results in males, females, participants over 44 years old, those with normal weight, and rural region individuals. In addition, there was a dose-response relationship between total PA and the risk of MACEs when total PA levels exceeded 17.60 MET-h/d. However, the results suggested that higher total PA might not be better when exploring the relationship with CVD because no further reductions were observed over ~31.78 MET-h/d.

Prospective cohort analysis in US elderly adults proved that PA, mainly walking, was beneficial in reducing the incidence of coronary heart disease and stroke among elderly adults (35). Additionally, a community-based sample of adults from the electronic Framingham Heart Study (eFHS) in the US reported that every increase of 1,000 steps in habitual physical activity was related to a 0.2% lower predicted CVD risk (*p*-value = 3.2 × 10^−4^) (36). A Chinese population-based prospective cohort study found a negative relationship between total PA and the risk of major vascular events, with an adjusted hazard ratio that compared the top (≥33.8 MET-h/d) with the bottom (≤9.1 MET-h/d) quintiles of PA at 0.77 (95% CI, 0.74~0.80) (18). Results in this study were in line with available evidence from previous studies, with an adjusted OR of 0.95 (95% CI, 0.93~0.98) in all participants.

However, the most recent global comparative study from 2018 indicates that one in four adults did not meet the World Health Organization (WHO) recommendations on PA to benefit from improving their health and wellbeing (37). This is mainly because of dramatic urbanization, reduction in PA in the workplace, changes in modes of transport, and other aspects of lifestyle, including in China (38, 39). Although we found that every four MET-h/d increase in PA was related to a lower risk of MACEs, our results might suggest a threshold association between total PA and the risk of MACEs. However, given that this study was based on cross-sectional data, we could not prove the causality. Some studies have reported that extreme endurance exercise may be detrimental to cardiovascular health (40), but the extent to which this may be relevant to the general population is unclear. A study based on objectively measured PA from a large population-based cohort study found no evidence of a threshold for the inverse association between objectively measured moderate, vigorous, and total PA and CVD (41). One potential reason might be related to the intensity of PA in total PA. A prospective cohort study of UK Biobank participants reported that a higher level of moderate-intensity PA was related to a lower risk of heart failure even beyond the current WHO PA recommendations. However, vigorous-intensity PA might have a lower potential risk reduction of heart failure when it exceeds the guidelines (42).

The benefits of PA have been proven at both biological and disease-specific levels. Biologically, many studies have demonstrated that PA can attenuate cardiovascular changes by improving the functional capacity of the cardiovascular system, cardiac function (43), and metabolism (44). A community-based study in the United States found that higher PA levels were associated with proportionally greater left ventricular mass and end-diastolic volume and lower resting heart rate among populations free of clinically apparent cardiovascular disease (43). In addition, some studies revealed that some cellular pathways (e.g., the insulin-like growth factor 1/PI3K/Akt pathway and nitric oxide signaling pathway) (45) and molecular mechanisms were associated with the positive effect of PA on cardiovascular disease. For example, experimental studies on rats revealed that the expression of cardiac heat shock protein 72 (HSP72) was robustly activated by exercise, promoting cardio-protection against ischemia-reperfusion injury (46).

Females appeared to have a more significant association between total PA per day and the risk of MACEs than males in this study, which was in line with a previous study (10, 18, 47). A meta-analysis of 21 prospective studies showed that moderate levels of occupational PA were related to more significant effects in females, especially coronary heart disease, compared with males (10). One possible explanation for the difference between males and females is that the women's reported physical activity may have occurred against a higher “background” level of activity in terms of routine household tasks (e.g., preparing meals, doing laundry, and light housework) and caring duties (47). A multistage study in Australia reported that men aged 40–65 years old spend 3.96 h on a usual weekday and 4.93 h on a typical weekend day in sedentary leisure behavior (e.g., watching television and using a computer at home), which is higher than their female counterparts (48). These differing “background” levels of light activity and sedentary behavior may be critical regarding long-term health outcomes (49).

In addition, we observed a difference in age groups, with participants over 45 years showing a beneficial relationship between total PA per day and the risk of MACE, which was in line with a prior study. A study based on the European Prospective Investigation into Cancer (EPIC) Norfolk prospective population study reported that there was a significant inverse association between PA and the risk of CVD when comparing individuals with the highest level of PA to inactive people in the elderly (>65 years) and people aged 55–65 years old (50). In the BMI groups, we found an association between total PA per day and a lower risk of MACEs in those participants with average weight after adjusting for sociodemographic factors and lifestyle factors. Obese individuals have lower relative muscle strength than non-obese individuals and have an increased risk of musculoskeletal injury/pathology (51). A previous study reported that PA did not eliminate the risk of CVD associated with elevated BMI. However, physical activity attenuated the increased risk of obesity in relatively healthy populations (52). Furthermore, in the exploratory analysis, we only observed the association between total PA per day and a lower risk of MACE in the individuals who did not have diabetes but were not individuals with diabetes. The results were inconsistent with the current study. A population-based cohort of patients with type 2 diabetes reported that the active patient (obtained from the primary care records and evaluated by nurse practitioners) had a 29% lower risk of CVD events than the inactive group (53). The inconsistency in the results might have the following reasons: firstly, the outcomes of CVD and diabetes were self-reported. Due to the lower access to health services and chronic disease management levels in northwest China, the overall prevalence of self-reported diabetes and CVD was low in this cohort's baseline survey (22). Besides, this study is a cross-sectional design, which means it is difficult to explain the temporal of diabetes and CVD.

The major strength of this study is the large population sample recruited from Shaanxi Province in northwest China. We collected a sizable number of outcomes to investigate the relationship between total PA and the risk of MACEs. In addition, we quantified the amount of PA and assessed the risk of MACEs with which it was associated. Moreover, we examined the associations by adjusting for a comprehensive list of confounders, including sociodemographic factors and lifestyle factors, to reduce the potential confounding bias.

This study also had several limitations. First, this cross-sectional study could not prove the causality between PA and the risk of MACEs and had the risk of reverse causality between them. The objective measurement or continuous monitoring of PA levels between exposure and outcome would allow for more accurate conclusions and is a focus of future work. Second, we used a questionnaire to collect data about daily activity. Due to differential measurement errors of self-reported questionnaires and incomplete coverage of PA types, there was uncertainty about the strength of this association (41). A study from a nationally representative sample of United States adults found that MVPA measured using accelerometers showed a stronger relationship with physiological and anthropometric biomarkers than self-reported MVPA (54). In addition, a multicenter study involving 10 regional test centers throughout Norway reported that the correlation coefficients between the International Physical Activity Questionnaire and objectively measured PA (by accelerometers) ranged from 0.20 to 0.46. Higher activity and intensity levels might be related to the increased difference between self-reported and accelerometer-measured MVPA (55). Thus, misreporting activity levels might have led to potential bias in the relationship between PA and the risk of MACE compared with objective PA measurement. However, for our study's large population, self-reported questionnaires still have some advantages, e.g., they are easy to manage during the data collection, are low-cost, and can collect detailed information on the activities performed (56). In addition, the primary outcome of this analysis was also collected by questionnaire, which cannot fully reflect the clinical outcomes of major cardiovascular events. Third, although we adjusted for confounders among sociodemographic and lifestyle factors, there might have still been residual confounding because of unknown and unmeasured factors (e.g., lipids, medication history, and the use of pharmacological treatments) or bias (e.g., recall bias). Thus, future research should focus on objective indicators to evaluate the effect of PA on CVD, such as cardiorespiratory fitness (57) and cardio-metabolic risk biomarkers (58). Finally, given that the mean age in this study was 50.15 years, the findings might not be generalizable to younger populations.

Controlling the lifestyle of CVD, including diet and physical activity, and reducing their geographical inequity should be critical points in addressing the daunting CVD burden in China (59). However, compared with central and eastern China, the region of northwest China had low economic and medical levels and unhealthy lifestyle behavior, which were the influence factors in the prevention of CVD (22). Our study results might help to give a clue in developing regional CVD prevention guidelines. To be more practical, we observed each four MET-h/d higher activity (~1 h of brisk walking per day) was associated with a 5% lower risk of MACE. Further research is needed to explore the causality between PA and the incidence of CVD in northwest China.

## Conclusions

In conclusion, higher levels of total PA were related to lower risks of MACEs in adults from northwest China. In addition, there was a “U”-shaped dose-response relationship between total PA and the risk of MACEs when total PA exceeded 17.60 MET-h/d. Future prospective studies should focus on device-measured PA (e.g., accelerometers or pedometers) or domain-specific PA (e.g., leisure-time PA and occupational PA), providing robust evidence to develop PA guidelines as an effective intervention strategy for CVD in the Chinese population.

## Data availability statement

The raw data supporting the conclusions of this article will be made available by the authors, without undue reservation.

## Ethics statement

The studies involving human participants were reviewed and approved by Human Research Ethics Committee of Xi'an Jiaotong University. The patients/participants provided their written informed consent to participate in this study.

## Members of the Regional Ethnic Cohort Study Collaborative Group

**Leadership:** Hong Yan (PI), Xinhua Wang (PI in Gansu Province), Jianghong Dai (PI in Xinjiang Province), Yuhong Zhang (PI in Ningxia), Xiaojie Wang (PI in Qinghai Province).

**Regional Co-ordinating Centers: School of Public Health, Xi'an Jiaotong University:** Shaonong Dang, Yuxue Bi, Lingxia Zeng, Quanli Wang, Qiang Li, Yuan Shen, Yaling Zhao, Leilei Pei, Fangyao Chen, Yijun Kang, Shengbin Xiao, Chao Li, Yue Cheng, Pengfei Qu, Baibing Mi. **Xi'an Jiaotong University Global Health Institute:** Youfa Wang. **The First Affiliated Hospital of Xi'an Jiaotong University:** Bingyin Shi, Qiumin Qu, Xinjun Lei. **The Second Affiliated Hospital of Xi'an Jiaotong University:** Zongfang Li. **Hospital of Stomatology Xi'an Jiaotong University:** Bofeng Zhu. **School of Mathematics and Statistics, Xi'an Jiaotong University:** Jian Sun, Huibin Li. **School of Management, Xi'an Jiaotong University:** Dehai Di. **Shaanxi Provincial CDC:** Feng Liu. **Taizhou Institute of Health Sciences, Fudan University:** Yanfeng Jiang, Tai Zhang, Xi Sun. **Baotou Medical College:** Suhua wang, Rui Qiao, Yuhang Zhao. **Gansu Provincial CDC:** Jianyun Sun, Tingcai Wang, Xiaolan Ren, Jing Zhang, Hupeng He, Lijuan Chen, Guihang Song, Shuyu Liu, Weitao Chen. **School of Public Health, Lanzhou University:** Wenlong Gao, Xiaoning Liu. **Research Institute of Xinjiang Chinese Medicine:** Fengsen Li, Zhanjun Shu, Qi Sun. **Urumqi Municipal CDC:** Baoling Rui, Gaofeng Sun, Qin Qin. **Xinjiang Medical University:** Jinfeng Ma, Pelton Mijiti. **Changji CDC:** Jun Yang, Xiulan Kang, Jie Mi. **Ningxia CDC:** Jianhua Zhao, Yin'e Zhang, Jiancai Du, Shaoning Ma. **Ningxia Medical University:** Yi Zhao, Lan Liu, Faxuan Wang, Yajuan Zhang, Yu Zhao. **Qinghai Provincial Hospital of Cardio-cerebrovascular Diseases:** Tianyi Wu, Shiming Liu, Fengyun Liu, Deng Geng, Yuxian Li, Jialin Wen. **Qinghai Provincial CDC:** Minru Zhou, Fuchang Ma, Zhihua Xu, Xiaoping Li, Qiongyue Sha, Ji Xing, Ji Che, Qi Song, Shengjin Zhang.

**Third-Party Medical Laboratories: Shenzhen Huada Gene Research Institute:** Bo Li, Tao Li, Wei Zhang, Xiaoyun Huang, Jiayu Chen, Xinyi Shuai. **Beijing Baidu Netcom Technology Co., Ltd.:** Weijie Gan, Jian He, Qingqing Li, Wenzhi Huang, Gang Zeng.

## Author contributions

YW and BM designed the study. YW, HL, and BM designed the analytical strategy and helped to interpret the findings and wrote the paper. BM had primary responsibility for the final content. DH, BZ, YL, KX, SC, YH, JL, LZ, HY, and SD provided additional interpretation of the data and revisions to the manuscript. All authors have read and approved the final manuscript.

## Funding

This study was funded by the National Key R&D Program of China (Grant Nos. 2017YFC0907200 and 2017YFC09072010), the National Natural Science Foundation of China (Grant No. 82103944), and the Natural Science Basic Research Plan of the Shaanxi Province (Grant No. 2020JQ-090). The funders/sponsors were not involved in the work.

## Conflict of interest

The authors declare that the research was conducted in the absence of any commercial or financial relationships that could be construed as a potential conflict of interest.

## Publisher's note

All claims expressed in this article are solely those of the authors and do not necessarily represent those of their affiliated organizations, or those of the publisher, the editors and the reviewers. Any product that may be evaluated in this article, or claim that may be made by its manufacturer, is not guaranteed or endorsed by the publisher.
