# Supplementary material for: Association between physical activity and major adverse cardiovascular events in northwest China: A cross-sectional analysis from the Regional Ethnic Cohort Study
Source: Front Public Health. 2022 Nov 17;10:1025670. doi: 10.3389/fpubh.2022.1025670 (PMC9713839; doi:10.3389/fpubh.2022.1025670)
Supplement: Supplementary file 1 [file Data_Sheet_1.docx]

Supplementary Material

# Supplementary Figures and Tables

## Supplementary Tables

**Supplementary Table 1.** Physical Activity Types, MET Values, Codes and Intensity Categories ^a^

| **Category** | **Activity type** | **Intensity** | **MET** | **Codes** |
| --- | --- | --- | --- | --- |
| Physical activity at work | Sedentary work | Low | 1.8 | Mean of 11580, 11585, and 11590 |
|  | standing work | Moderate | 3.8 | Mean of 11610 and 11630 |
|  | General manual work | Moderate | 4.5 | 11476 |
|  | Heavy manual work | Vigorous | 6.5 | 11477 |
| Farming season | Manual work in the farming season | Vigorous | 6.3 | Mean of 11145 and 11146 |
|  | Semi-mechanized work in the farming season | Moderate | 3.4 | Mean of 11146 and 11147 |
|  | Fully mechanized work in the farming season | Low | 2.4 | Mean of 11147 and 11170 |
|  | slack season farming work | Low | 2.0 | 11147 |
|  | Farming season Vigorous work | Vigorous | 7.8 | 11145 |
| Commuting | Walking | Moderate | 4.0 | 17270 |
|  | Bicycle | Vigorous | 6.8 | 01011 |
|  | Motorbike | Moderate | 3.5 | 16030 |
|  | Private car or taxi | Low | 1.9 | Mean of 16010 and 16015 |
|  | Public transportation | Low | 1.7 | 16016 |
| Housework | Household activity | Low | 2.8 | Mean of 05030^#^, 05040^#^, 05035, 05055, 05070, 05090^#^, 05092^#^, 05184, 05197, and 05200 |
| Vigorous physical activity | Vigorous physical activity | vigorous | 8.0 | Mean of PAD615 and PAD660 ^b^ |
| Physical exercise | Tai-Chi/qigong/leisure walking/Yoga | Moderate | 3.3 | Mean of 15670 and 17160 |
|  | long distance running/aerobics | Vigorous | 7.4 | Mean of 12020,12150 and 03015 |
|  | Ball games | Moderate | 5.5 | Mean of 15020^#^, 15030^#^, 15055, 15080, 15090, 15255, 15605^#,^ 15610^#^, 15652, 15660, 15675, 15710^#^, and 15711^#^ |
|  | Swimming | Vigorous | 7.2 | Mean of 18230, 18240, and 18310 |
|  | Brisk walking/gymnastics/folk dancing | Moderate | 4.2 | Mean of 03025, 15300, and 17200 |
|  | Other exercises, e.g. mountain walking, home exercise and rope jumping | Moderate | 5.9 | Mean of 02010, 02064, 04001, 04100, 15110^#^, 15120^#^, 15200, 15240, 15310, 15425^#^, 15430^#^, 15537, 15550^##^, 15551^##^, 15552^##^, 15580, 15590, 15730, 15732^##^, 15733^##^, 15734^##^ and 19030 |
| Sedentary activity | Watching TV | Low | 1.2 | Mean of 07010 and 07020 |
|  | Phones and pads | Low | 1.7 | Mean of 09050 and 09055 |
|  | Playing chess/ cards/ mahjong | Low | 1.5 | Mean of 09000, 09010 and 09013 |
|  | Reading books and documentation | Low | 1.5 | Mean of 09030,09070,07070 |
|  | Computers / Writing / reading documentation | Low | 1.5 | Mean of 09015, 09040, 09045, 09050^#^, 09055^#^, 09060,09075,09070, 07050,07070 |
|  | All sedentary activities (Unclassified) | Low | 1.5 | Mean of 5 categories of sedentary activity |
| Sleep | Sleep duration | Low | 1.0 | 07030 |

MET, Metabolic equivalent task.

^a^ Based on the 2011 Compendium of Physical Activities: a second update of codes and MET values. Ainsworth BE, et al. Medicine and Science in Sports and Exercise, 2011;43(8):1575-1581.

^#^ Assigned 1/2 weight in calculating the mean MET value because the connecting two items represent one type of activity.

^##^ Assigned 1/3 weight in calculating the mean MET value because the connecting three items represent one type of activity.

^b^ Based on NHANES website: https://wwwn.cdc.gov/Nchs/Nhanes/2011-2012/PAQ_G.htm#PAQ605.

**Supplementary Table 2.** Comparison of participant characteristics between the analytic and excluded samples ^a^

| **Characteristics** | **Analytic sample** | **Excluded sample** | ***p*-value** |
| --- | --- | --- | --- |
| **No. of participants** | 34 597 | 13 428 |  |
| **Mean ± SD ^b^** |  |  |  |
| **Age, years** | 50.15±12.81 | 46.82±14.86 | <0.001 |
| **BMI, kg/m^2^** | 23.80±3.40 | 23.93±3.89 | 0.001 |
| **Physical activity–related factors** |  |  |  |
| Total physical activity, MET-h/d | 17.60±11.50 | 25.53±18.79 | <0.001 |
| Sedentary leisure time, h/d | 2.99±1.72 | 4.35±3.14 | <0.001 |
| ***n* (%)** |  |  |  |
| **Demographic factors** |  |  |  |
| Female | 20 932 (60.50) | 8 496 (63.27) | <0.001 |
| Rural | 22 790 (65.87) | 6 088 (51.58) | <0.001 |
| Han Chinese ethnicity | 34 198 (99.14) | 13 200 (98.95) | <0.001 |
| **Socioeconomic and lifestyle factors** |  |  |  |
| Middle school | 13 647 (39.60) | 4 073 (30.63) | <0.001 |
| Married | 30 285 (88.04) | 11 555 (87.27) | <0.001 |
| Household annual income ≥100 000 ¥/year ^c^ | 4 631 (14.97) | 2 107 (18.54) | <0.001 |
| Current smoker | 7 163 (20.91) | 2 575 (19.63) | <0.001 |
| Current drinker | 22 981 (66.72) | 4 881 (37.00) | <0.001 |
| **Self-reported conditions** |  |  |  |
| Good status of health | 11 261 (32.72) | 4 126 (31.96) | <0.001 |
| Hypertension | 4 164 (37.50) | 1 895 (33.58) | <0.001 |
| Diabetes | 997 (9.47) | 659 (12.17) | <0.001 |
| MACE | 1 048 (3.24) | 806 (6.84) | <0.001 |

MET-h/d, metabolic equivalents of task per hour per day; SD, standard deviation; BMI, body mass index (calculated as weight in kilograms divided by height in meters squared); MACE, major adverse cardiovascular event.

^a^ For some variables, the sum of categories was not equal to the total due to missing data.

^b^ Continuous variables were presented as Mean (SD) and categorical variables were presented as n (%).

^c^ RMB (yuan) was used to estimate household annual income.

**Supplementary Table 3.** Associations between total physical activity and the subtype of MACE in all participants

|  | **Sample Size** | **Prevalence, *n* (%)** | **Model 1 (crude)** | ***p*-value** | **Model 2 ^a^** | ***p*-value** | **Model 3 ^b^** | ***p*-value** |
| --- | --- | --- | --- | --- | --- | --- | --- | --- |
|  |  |  | **OR (95% CI)** | | | | | |
| **Acute Myocardial infarction** |  |  |  |  |  |  |  |  |
| Total physical activity (MET-h/d) | 32 182 | 241 (0.74) | 0.85 (0.80,0.90) | <0.001 | 0.91 (0.86,0.97) | 0.001 | 0.91 (0.86,0.97) | 0.002 |
| Total physical activity (four categories) ^c^ |  |  |  |  |  |  |  |  |
| <9.0 | 7 937 | 102 (1.29) | 1.00 [Reference] |  | 1.00 [Reference] |  | 1.00 [Reference] |  |
| 9.0-15.2 | 7 958 | 64 (0.80) | 0.62 (0.46,0.85) | 0.003 | 0.89 (0.64,1.24) | 0.507 | 0.96 (0.69,1.35) | 0.834 |
| 15.3-23.2 | 8 101 | 38 (0.47) | 0.37 (0.25,0.53) | <0.001 | 0.75 (0.50,1.10) | 0.143 | 0.73 (0.48,1.10) | 0.129 |
| ≥23.3 | 8 186 | 37 (0.45) | 0.35 (0.24,0.51) | <0.001 | 0.52 (0.34,0.79) | 0.003 | 0.52 (0.34,0.82) | 0.004 |
| **Stroke/minor stroke** |  |  |  |  |  |  |  |  |
| Total physical activity (MET-h/d) | 32 279 | 832 (2.58) | 0.90 (0.88,0.93) | <0.001 | 0.96 (0.94,0.99) | 0.006 | 0.96 (0.94,0.99) | 0.007 |
| Total physical activity (four categories) ^c^ |  |  |  |  |  |  |  |  |
| <9.0 | 7 967 | 329 (4.13) | 1.00 [Reference] |  | 1.00 [Reference] |  | 1.00 [Reference] |  |
| 9.0-15.2 | 7 998 | 207 (2.59) | 0.62 (0.52,0.74) | <0.001 | 1.00 (0.83,1.20) | 0.998 | 1.04 (0.85,1.26) | 0.723 |
| 15.3-23.2 | 8 105 | 140 (1.73) | 0.41 (0.33,0.50) | <0.001 | 0.93 (0.75,1.15) | 0.524 | 0.94 (0.75,1.17) | 0.563 |
| ≥23.3 | 8 209 | 156 (1.90) | 0.45 (0.37,0.55) | <0.001 | 0.72 (0.58,0.88) | 0.002 | 0.72 (0.58,0.89) | 0.003 |

MACE, major adverse cardiovascular event; MET-h/d, metabolic equivalents of task per hour per day; OR, odds ratio; CI, confidence interval.

^a^ Model 2 adjusted for age, sex, study location, household annual income, and education.

^b^ Model 3 additionally adjusted for smoking, alcohol consumption, sedentary leisure time, fruit intake, and self-reported general health status.

^c^ The value of METs was categorized into four groups by quartile: 9.0 (quartile1), 15.2 (quartile2), 23.2 (quartile3).

**Supplementary Table 4.** Associations between total physical activity and the prevalence of MACE in different sex groups

|  | **Sample Size** | **Prevalence, *n* (%)** | **Model 1 (crude)** | ***p*-value** | **Model 2 ^a^** | ***p*-value** | **Model 3 ^b^** | ***p*-value** |
| --- | --- | --- | --- | --- | --- | --- | --- | --- |
|  |  |  | **OR (95% CI)** | | | | | |
| **Sex groups** |  |  |  |  |  |  |  |  |
| **Male** |  |  |  |  |  |  |  |  |
| Total physical activity (MET-h/d) | 12 717 | 498 (3.92) | 0.89 (0.86,0.92) | <0.001 | 0.96 (0.93,0.99) | 0.016 | 0.97 (0.94,1.00) | 0.045 |
| Total physical activity (four categories) ^c^ |  |  |  |  |  |  |  |  |
| <9.0 | 3 221 | 221 (6.86) | 1.00 [Reference] |  | 1.00 [Reference] |  | 1.00 [Reference] |  |
| 9.0-15.2 | 2 717 | 103 (3.79) | 0.54 (0.42,0.68) | <0.001 | 0.95 (0.73,1.23) | 0.686 | 1.02 (0.78,1.33) | 0.890 |
| 15.3-23.2 | 3 277 | 72 (2.20) | 0.31 (0.23,0.40) | <0.001 | 0.85 (0.64,1.13) | 0.269 | 0.88 (0.65,1.19) | 0.401 |
| ≥23.3 | 3 502 | 102 (2.91) | 0.41 (0.32,0.52) | <0.001 | 0.70 (0.54,0.91) | 0.009 | 0.73 (0.56,0.97) | 0.028 |
| **Female** |  |  |  |  |  |  |  |  |
| Total physical activity (MET-h/d) | 19 621 | 550 (2.80) | 0.89 (0.86,0.92) | <0.001 | 0.94 (0.91,0.98) | 0.001 | 0.93 (0.90,0.97) | 0.001 |
| Total physical activity (four categories) |  |  |  |  |  |  |  |  |
| <9.0 | 4 771 | 199 (4.17) | 1.00 [Reference] |  | 1.00 [Reference] |  | 1.00 [Reference] |  |
| 9.0-15.2 | 5 297 | 162 (3.06) | 0.73 (0.59,0.90) | 0.003 | 0.98 (0.78,1.22) | 0.836 | 1.02 (0.81,1.28) | 0.874 |
| 15.3-23.2 | 4 841 | 101 (2.09) | 0.49 (0.39,0.63) | <0.001 | 0.90 (0.70,1.17) | 0.438 | 0.89 (0.68,1.16) | 0.396 |
| ≥23.3 | 4 712 | 88 (1.87) | 0.44 (0.34,0.57) | <0.001 | 0.63 (0.47,0.83) | 0.001 | 0.62 (0.46,0.82) | 0.001 |

MACE, major adverse cardiovascular event; MET-h/d, metabolic equivalents of task per hour per day; OR, odds ratio; CI, confidence interval.

^a^ Model 2 adjusted for age, sex, study location, household annual income, and education.

^b^ Model 3 additionally adjusted for smoking, alcohol consumption, sedentary leisure time, fruit intake, and self-reported general health status.

^c^ The value of METs was categorized into four groups by quartile: 9.0 (quartile1), 15.2 (quartile2), 23.2 (quartile3).

**Supplementary Table 5.** Associations between total physical activity and the prevalence of MACE in different age groups

|  | **Sample Size** | **Prevalence, *n* (%)** | **Model 1 (crude)** | ***p*-value** | **Model 2 ^a^** | ***p*-value** | **Model 3 ^b^** | ***p*-value** |
| --- | --- | --- | --- | --- | --- | --- | --- | --- |
|  |  |  | **OR (95% CI)** | | | | | |
| **Age groups** |  |  |  |  |  |  |  |  |
| **18-44** |  |  |  |  |  |  |  |  |
| Total physical activity (MET-h/d) | 10 111 | 17 (0.17) | 0.98 (0.81,1.18) | 0.819 | 1.03 (0.88,1.21) | 0.697 | 1.04 (0.89,1.21) | 0.653 |
| Total physical activity (four categories) ^c^ |  |  |  |  |  |  |  |  |
| <9.0 | 1 147 | 3 (0.26) | 1.00 [Reference] |  | 1.00 [Reference] |  | 1.00 [Reference] |  |
| 9.0-15.2 | 2 588 | 7 (0.27) | 0.95 (0.27,3.39) | 0.937 | 2.57 (0.73,9.05) | 0.143 | 2.56 (0.80,8.22) | 0.113 |
| 15.3-23.2 | 3 783 | 2 (0.05) | 0.22 (0.04,1.10) | 0.065 | 1.27 (0.28,5.77) | 0.755 | 0.91 (0.19,4.23) | 0.899 |
| ≥23.3 | 2 593 | 5 (0.19) | 0.69 (0.18,2.66) | 0.595 | 1.73 (0.40,7.46) | 0.462 | 1.95 (0.50,7.64) | 0.335 |
| **45-59** |  |  |  |  |  |  |  |  |
| Total physical activity (MET-h/d) | 13 934 | 338 (2.43) | 0.96 (0.92,0.99) | 0.018 | 0.93 (0.90,0.97) | 0.001 | 0.93 (0.89,0.97) | <0.001 |
| Total physical activity (four categories) |  |  |  |  |  |  |  |  |
| <9.0 | 3 577 | 111 (3.10) | 1.00 [Reference] |  | 1.00 [Reference] |  | 1.00 [Reference] |  |
| 9.0-15.2 | 3 350 | 78 (2.33) | 0.75 (0.56,1.00) | 0.050 | 0.83 (0.61,1.13) | 0.232 | 0.85 (0.62,1.16) | 0.306 |
| 15.3-23.2 | 2 980 | 60 (2.01) | 0.64 (0.47,0.88) | 0.007 | 0.71 (0.51,0.99) | 0.042 | 0.70 (0.50,1.00) | 0.047 |
| ≥23.3 | 4 027 | 89 (2.21) | 0.71 (0.53,0.94) | 0.016 | 0.60 (0.45,0.81) | 0.001 | 0.59 (0.43,0.81) | 0.001 |
| **≥60** |  |  |  |  |  |  |  |  |
| Total physical activity (MET-h/d) | 8 293 | 693 (8.36) | 0.95 (0.92,0.98) | 0.001 | 0.94 (0.91,0.97) | <0.001 | 0.95 (0.92,0.98) | 0.001 |
| Total physical activity (four categories) |  |  |  |  |  |  |  |  |
| <9.0 | 3 268 | 306 (9.36) | 1.00 [Reference] |  | 1.00 [Reference] |  | 1.00 [Reference] |  |
| 9.0-15.2 | 2 076 | 180 (8.67) | 0.92 (0.76,1.12) | 0.396 | 0.98 (0.80,1.20) | 0.836 | 1.03 (0.84,1.27) | 0.747 |
| 15.3-23.2 | 1 355 | 111 (8.19) | 0.87 (0.69,1.09) | 0.214 | 0.90 (0.72,1.14) | 0.401 | 0.93 (0.73,1.18) | 0.533 |
| ≥23.3 | 1 594 | 96 (6.02) | 0.62 (0.49,0.79) | <0.001 | 0.58 (0.46,0.75) | <0.001 | 0.60 (0.46,0.77) | <0.001 |

MACE, major adverse cardiovascular event; MET-h/d, metabolic equivalents of task per hour per day; OR, odds ratio; CI, confidence interval.

^a^ Model 2 adjusted for age, sex, study location, household annual income, and education.

^b^ Model 3 additionally adjusted for smoking, alcohol consumption, sedentary leisure time, fruit intake, and self-reported general health status.

^c^ The value of METs was categorized into four groups by quartile: 9.0 (quartile1), 15.2 (quartile2), 23.2 (quartile3).

**Supplementary Table 6.** Associations between total physical activity and the prevalence of MACE in different BMI groups ^a^

|  | **Sample Size** | **Prevalence, *n* (%)** | **Model 1 (crude)** | ***p*-value** | **Model 2 ^b^** | ***p*-value** | **Model 3 ^c^** | ***p*-value** |
| --- | --- | --- | --- | --- | --- | --- | --- | --- |
|  |  |  | **OR (95% CI)** | | | | | |
| **BMI groups** |  |  |  |  |  |  |  |  |
| **Normal weight** |  |  |  |  |  |  |  |  |
| Total physical activity (MET-h/d) | 17 632 | 495 (2.81) | 0.88 (0.85,0.92) | <0.001 | 0.95 (0.92,0.99) | 0.005 | 0.95 (0.92,0.99) | 0.007 |
| Total physical activity (four categories) ^d^ |  |  |  |  |  |  |  |  |
| <9.0 | 4 379 | 207 (4.73) | 1.00 [Reference] |  | 1.00 [Reference] |  | 1.00 [Reference] |  |
| 9.0-15.2 | 4 404 | 128 (2.91) | 0.60 (0.48,0.76) | <0.001 | 1.06 (0.84,1.35) | 0.612 | 1.16 (0.90,1.48) | 0.244 |
| 15.3-23.2 | 4 408 | 73 (1.66) | 0.34 (0.26,0.45) | <0.001 | 0.86 (0.64,1.15) | 0.301 | 0.87 (0.65,1.18) | 0.376 |
| ≥23.3 | 4 441 | 87 (1.96) | 0.40 (0.31,0.52) | <0.001 | 0.64 (0.48,0.85) | 0.002 | 0.66 (0.49,0.89) | 0.006 |
| **Overweight range** |  |  |  |  |  |  |  |  |
| Total physical activity (MET-h/d) | 11 091 | 410 (3.70) | 0.91 (0.87,0.94) | <0.001 | 0.97 (0.94,1.01) | 0.138 | 0.96 (0.93,1.00) | 0.074 |
| Total physical activity (four categories) |  |  |  |  |  |  |  |  |
| <9.0 | 2 781 | 164 (5.90) | 1.00 [Reference] |  | 1.00 [Reference] |  | 1.00 [Reference] |  |
| 9.0-15.2 | 2 706 | 97 (3.58) | 0.59 (0.46,0.77) | <0.001 | 0.85 (0.65,1.12) | 0.251 | 0.91 (0.68,1.20) | 0.500 |
| 15.3-23.2 | 2 750 | 70 (2.55) | 0.42 (0.32,0.56) | <0.001 | 0.84 (0.62,1.14) | 0.268 | 0.83 (0.61,1.14) | 0.255 |
| ≥23.3 | 2 854 | 79 (2.77) | 0.46 (0.35,0.60) | <0.001 | 0.72 (0.54,0.97) | 0.031 | 0.68 (0.50,0.93) | 0.014 |
| **Obesity range** |  |  |  |  |  |  |  |  |
| Total physical activity (MET-h/d) | 3 296 | 137 (4.16) | 0.87 (0.81,0.93) | <0.001 | 0.93 (0.87,0.99) | 0.036 | 0.94 (0.88,1.01) | 0.088 |
| Total physical activity (four categories) |  |  |  |  |  |  |  |  |
| <9.0 | 796 | 48 (6.03) | 1.00 [Reference] |  | 1.00 [Reference] |  | 1.00 [Reference] |  |
| 9.0-15.2 | 815 | 40 (4.91) | 0.81 (0.52,1.24) | 0.325 | 1.13 (0.72,1.78) | 0.601 | 1.09 (0.68,1.75) | 0.730 |
| 15.3-23.2 | 847 | 26 (3.07) | 0.50 (0.31,0.81) | 0.005 | 1.05 (0.63,1.75) | 0.854 | 1.14 (0.67,1.94) | 0.638 |
| ≥23.3 | 838 | 23 (2.74) | 0.44 (0.27,0.74) | 0.002 | 0.68 (0.40,1.16) | 0.155 | 0.78 (0.45,1.35) | 0.381 |

BMI, body mass index (calculated as weight in kilograms divided by height in meters squared); MACE, major adverse cardiovascular event; MET-h/d, metabolic equivalents of task per hour per day; OR, odds ratio; CI, confidence interval.

^a^ BMI was categorized into three groups: normal weight (BMI<24 kg/m^2^), overweight (24 kg/m^2^≤BMI<28 kg/m^2^), and obesity (BMI≥28 kg/m^2^).

^b^ Model 2 adjusted for age, sex, study location, household annual income, and education.

^c^ Model 3 additionally adjusted for smoking, alcohol consumption, sedentary leisure time, fruit intake, and self-reported general health status.

^d^ The value of METs was categorized into four groups by quartile: 9.0 (quartile1), 15.2 (quartile2), 23.2 (quartile3).

**Supplementary Table 7.** Associations between total physical activity and the prevalence of MACE in different region groups

|  | **Sample Size** | **Prevalence, *n* (%)** | **Model 1 (crude)** | ***p*-value** | **Model 2 ^a^** | ***p*-value** | **Model 3 ^b^** | ***p*-value** |
| --- | --- | --- | --- | --- | --- | --- | --- | --- |
|  |  |  | **OR (95% CI)** | | | | | |
| **Region groups** |  |  |  |  |  |  |  |  |
| **Urban region** |  |  |  |  |  |  |  |  |
| Total physical activity (MET-h/d) | 10 838 | 118 (1.08) | 0.80 (0.72,0.88) | <0.001 | 1.00 (0.92,1.10) | 0.932 | 1.00 (0.89,1.12) | 0.956 |
| Total physical activity (four categories) ^c^ |  |  |  |  |  |  |  |  |
| <9.0 | 1 016 | 40 (3.94) | 1.00 [Reference] |  | 1.00 [Reference] |  | 1.00 [Reference] |  |
| 9.0-15.2 | 2 978 | 28 (0.94) | 0.23 (0.14,0.38) | <0.001 | 0.71 (0.39,1.29) | 0.262 | 0.93 (0.46,1.86) | 0.834 |
| 15.3-23.2 | 4 369 | 31 (0.71) | 0.18 (0.11,0.28) | <0.001 | 1.04 (0.59,1.86) | 0.883 | 1.14 (0.57,2.28) | 0.705 |
| ≥23.3 | 2 473 | 19 (0.77) | 0.19 (0.11,0.33) | <0.001 | 0.79 (0.40,1.56) | 0.500 | 0.74 (0.32,1.75) | 0.497 |
| **Rural region** |  |  |  |  |  |  |  |  |
| Total physical activity (MET-h/d) | 21 502 | 930 (4.33) | 0.92 (0.90,0.94) | <0.001 | 0.95 (0.93,0.98) | <0.001 | 0.95 (0.93,0.98) | <0.001 |
| Total physical activity (four categories) |  |  |  |  |  |  |  |  |
| <9.0 | 6 976 | 380 (5.45) | 1.00 [Reference] |  | 1.00 [Reference] |  | 1.00 [Reference] |  |
| 9.0-15.2 | 5 036 | 237 (4.71) | 0.86 (0.73,1.01) | 0.071 | 1.01 (0.85,1.20) | 0.938 | 1.04 (0.87,1.24) | 0.694 |
| 15.3-23.2 | 3 749 | 142 (3.79) | 0.68 (0.56,0.83) | <0.001 | 0.87 (0.71,1.06) | 0.171 | 0.87 (0.70,1.07) | 0.189 |
| ≥23.3 | 5 741 | 171 (2.98) | 0.53 (0.44,0.64) | <0.001 | 0.67 (0.55,0.81) | <0.001 | 0.67 (0.55,0.82) | <0.001 |

MACE, major adverse cardiovascular event; MET-h/d, metabolic equivalents of task per hour per day; OR, odds ratio; CI, confidence interval.

^a^ Model 2 adjusted for age, sex, study location, household annual income, and education.

^b^ Model 3 additionally adjusted for smoking, alcohol consumption, sedentary leisure time, fruit intake, and self-reported general health status.

^c^ The value of METs was categorized into four groups by quartile: 9.0 (quartile1), 15.2 (quartile2), 23.2 (quartile3).

**Supplementary Table 8.** Associations between total physical activity and MACE in diabetes participants and non-diabetes participants

|  | **Sample Size** | **Prevalence, *n* (%)** | **Model 1 (crude)** | ***p*-value** | **Model 2 ^a^** | ***p*-value** | **Model 3 ^b^** | ***p*-value** |
| --- | --- | --- | --- | --- | --- | --- | --- | --- |
|  |  |  | **OR (95% CI)** | | | | | |
| **Participants with diabetes** |  |  |  |  |  |  |  |  |
| Total physical activity (MET-h/d) | 797 | 84 (10.54) | 0.93 (0.85,1.01) | 0.083 | 0.99 (0.90,1.08) | 0.766 | 0.98 (0.90,1.08) | 0.745 |
| Total physical activity (four categories) ^c^ |  |  |  |  |  |  |  |  |
| <9.0 | 233 | 31 (13.30) | 1.00 [Reference] |  | 1.00 [Reference] |  | 1.00 [Reference] |  |
| 9.0-15.2 | 206 | 25 (12.14) | 0.90 (0.52,1.58) | 0.722 | 1.17 (0.64,2.12) | 0.617 | 1.02 (0.54,1.93) | 0.958 |
| 15.3-23.2 | 169 | 13 (7.69) | 0.55 (0.28,1.09) | 0.086 | 0.82 (0.41,1.65) | 0.581 | 0.82 (0.40,1.68) | 0.582 |
| ≥23.3 | 189 | 15 (7.94) | 0.57 (0.30,1.09) | 0.087 | 0.90 (0.45,1.81) | 0.777 | 0.84 (0.41,1.74) | 0.649 |
| **Participants without diabetes** |  |  |  |  |  |  |  |  |
| Total physical activity (MET-h/d) | 9 525 | 782 (8.21) | 0.95 (0.92,0.98) | <0.001 | 0.96 (0.93,0.98) | 0.002 | 0.96 (0.93,0.98) | 0.003 |
| Total physical activity (four categories) ^c^ |  |  |  |  |  |  |  |  |
| <9.0 | 3 058 | 322 (10.53) | 1.00 [Reference] |  | 1.00 [Reference] |  | 1.00 [Reference] |  |
| 9.0-15.2 | 2 361 | 179 (7.58) | 0.70 (0.58,0.84) | <0.001 | 0.92 (0.75,1.14) | 0.459 | 0.96 (0.78,1.19) | 0.725 |
| 15.3-23.2 | 2 109 | 136 (6.45) | 0.59 (0.48,0.72) | <0.001 | 0.94 (0.75,1.19) | 0.608 | 0.96 (0.75,1.22) | 0.729 |
| ≥23.3 | 1 997 | 145 (7.26) | 0.67 (0.54,0.82) | <0.001 | 0.69 (0.55,0.87) | 0.002 | 0.70 (0.55,0.89) | 0.004 |

MACE, major adverse cardiovascular event; MET-h/d, metabolic equivalents of task per hour per day; OR, odds ratio; CI, confidence interval.

^a^ Model 2 adjusted for age, sex, study location, household annual income, and education.

^b^ Model 3 additionally adjusted for smoking, alcohol consumption, sedentary leisure time, fruit intake, and self-reported general health status.

^c^ The value of METs was categorized into four groups by quartile: 9.0 (quartile1), 15.2 (quartile2), 23.2 (quartile3).

##
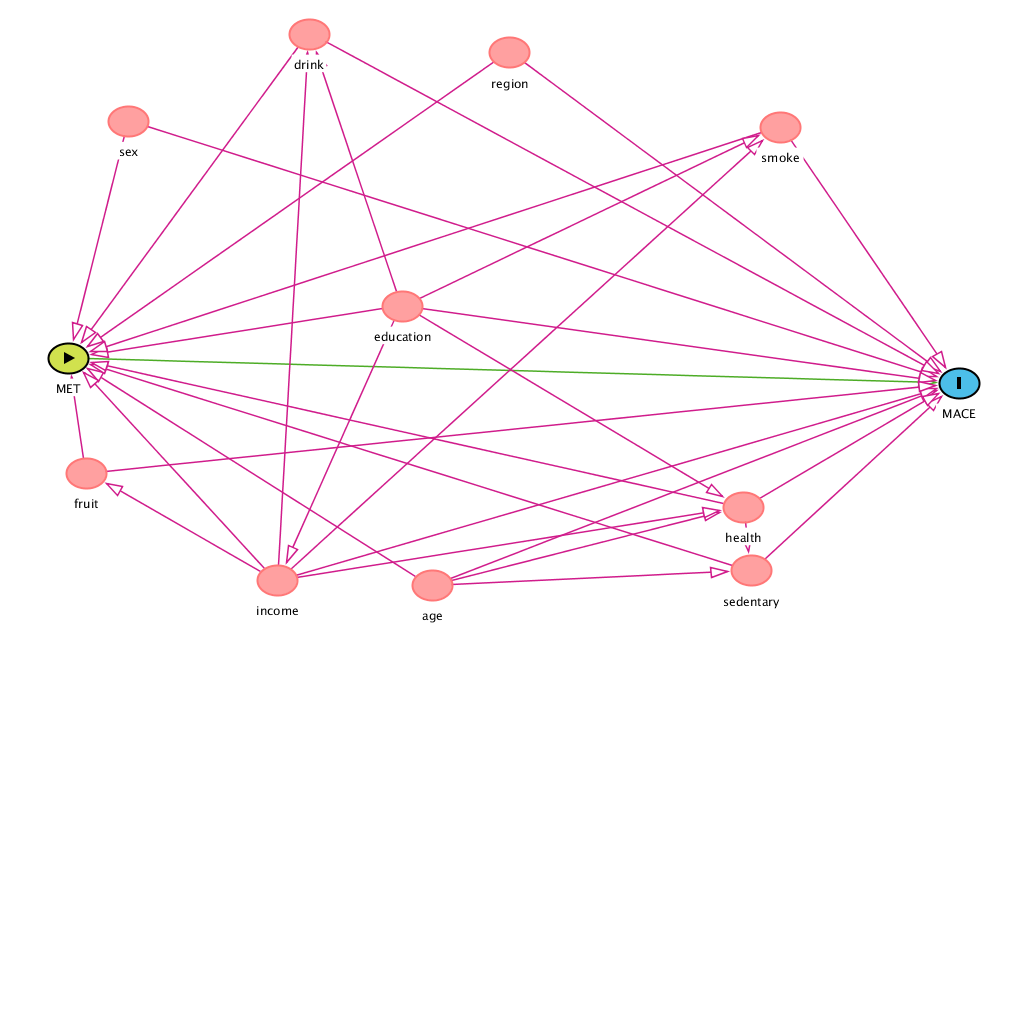
Supplementary Figures

**Supplementary Figure 1.** Directed acyclic graphs for the association between total physical activity and the risk of major adverse cardiovascular events, created with the help of DAGitty.net (http://dagitty.net/). Minimally sufficient adjustment set: age, sex, study location, household annual income, education, smoking, alcohol consumption, sedentary leisure time, fruit intake, and self-reported general health status.
